# BECLIN-1 is essential for the maintenance of gastrointestinal epithelial integrity by regulating endocytic trafficking, F-actin organization, and lysosomal function

**DOI:** 10.1080/27694127.2025.2484494

**Published:** 2025-04-03

**Authors:** Juliani Juliani, Sharon Tran, Tiffany J. Harris, Peter De Cruz, Sarah L. Ellis, Paul A. Gleeson, John M. Mariadason, Kinga Duszyc, Alpha S. Yap, Erinna F. Lee, Walter D. Fairlie

**Affiliations:** a Cell Death and Survival Laboratory, Olivia Newton-John Cancer Research Institute, Heidelberg, Australia; b School of Cancer Medicine, La Trobe University, Bundoora, Australia; c Department of Biochemistry and Chemistry, School of Agriculture, Biomedicine and Environment, La Trobe University, Bundoora, Australia; d La Trobe Institute for Molecular Science, La Trobe University, Bundoora, Australia; e Department of Gastroenterology, Austin Health, Melbourne, Australia; f Department of Medicine, Austin Health, The University of Melbourne, Melbourne, Australia; g Department of Biochemistry and Pharmacology and Bio21 Molecular Science and Biotechnology Institute, The University of Melbourne, Melbourne, Australia; h Institute for Molecular Bioscience, The University of Queensland, Brisbane, Australia

**Keywords:** F-actin, cytoskeleton, autophagy, ATG7, BECLIN-1, E-CADHERIN, endocytosis, OCCLUDIN, lysosome, intestinal homeostasis, IBD

## Abstract

Disrupted intestinal homeostasis and barrier function contribute to the development of diseases such as inflammatory bowel disease. BECLIN-1, a core component of two class III phosphatidylinositol 3 kinase complexes, has a dual role in autophagy and endocytic trafficking. Emerging evidence suggests that its endocytic trafficking function is essential for intestinal integrity. To investigate the fatal gastrointestinal phenotype observed in BECLIN-1 knockout mice, we used organoids derived from these animals to show that BECLIN-1 deletion disrupts the localization of CADHERIN1/ECADHERIN to adherens junctions and OCCLUDIN to tight junctions. Impaired cargo trafficking to the lysosome was also observed. Filamentous actin cytoskeleton also became disorganized though there were no changes in its spatial interaction with CATENIN BETA1/BETA-CATENIN nor in BETA-CATENIN localization. The trafficking defects were all less pronounced or absent in organoids lacking an autophagy-only regulator, ATG7, emphasizing BECLIN-1ʹs trafficking role in maintaining gut homeostasis and barrier function. These findings advance our understanding of epithelial dysfunction and the mechanisms underlying intestinal diseases.

## Introduction

The maintenance of intestinal homeostasis relies on a complex interplay of cellular processes that provide a protective barrier and ensure effective nutrient absorption and immune cell regulation [1,2]. Over the past two decades, research into the role of autophagy in gut health has grown significantly, stemming from landmark discoveries that uncovered polymorphisms in the core autophagy gene *ATG16L1* (autophagy-related-16-like 1) linked to Crohn’s disease [3–6]. This breakthrough, together with subsequent mechanistic studies, provided a deeper understanding of the genetic basis of inflammatory bowel disease (IBD) and highlighted the critical role autophagy plays in intestinal health [7,8]. Subsequently, a multitude of genetic models in which key autophagy regulators were modified has been generated to decipher the role of autophagy in intestinal epithelial cells (IECs) [1]. Specifically, these studies have elucidated the contribution of autophagy to immune responses in the gut [9,10], intestinal barrier integrity [11–14], gut microbiota biology [15,16], as well as the pathogenesis of intestinal diseases [17,18], and therapeutic potential for treating gut-related disorders [13,19].

BECLIN-1 is a core component of two class III phosphatidylinositol 3 kinase (PtdIns3K) complexes and acts as a signal integration hub with roles in both autophagy and endocytic trafficking [20]. Accordingly, BECLIN-1 has been implicated in the pathogenesis of numerous diseases, including various cancers (breast, ovarian, prostate, colorectal), Alzheimer’s disease, cystic fibrosis, sepsis, and others [20–28]. Germline deletion of BECLIN-1 results in an embryonic lethal phenotype in contrast to constitutive deletion of autophagy conjugation proteins such as ATG5 (autophagy-related 5) or ATG7, which result in neonatal lethality [22,29]. This disparity suggests an essential role for BECLIN-1 beyond autophagy during development.

In a recent study, we circumvented the embryonic lethality of germline BECLIN-1 deletion using a conditional BECLIN-1 knockout mouse model, which enabled inducible, whole body, and intestinal epithelial cell-specific BECLIN-1 deletion in adult mice. This led to a fatal enteritis phenotype that we attributed, using intestinal organoids, to defects in endocytic trafficking that resulted in mislocalization of the critical junctional protein, E-CADHERIN. This severe phenotype was not observed in equivalent studies involving deletion of the autophagy regulator ATG7, which is not associated with trafficking, underscoring a unique and essential function for BECLIN-1 in maintaining intestinal homeostasis [11,30]. Additional published reports have also implicated BECLIN-1 in maintaining intestinal homeostasis though these primarily focused on the colon and relied on autophagy-proficient experimental systems such as Tat-Beclin1 peptide treatment and *Becn1*^F121A^ mice [13,16].

In this study, we show that the role of BECLIN-1 in maintaining small intestinal integrity extends beyond the localization of E-CADHERIN at the adherens junctions (AJs) and is also critical in forming tight junctions (TJs). In addition, we show that its deletion leads to lysosomal dysfunction and cytoskeletal disruption, further compromising the epithelial barrier. Combined, these data provide a comprehensive understanding of how BECLIN-1 supports intestinal homeostasis by coordinating junctional integrity, endocytic trafficking, and cellular architecture to preserve the epithelial barrier.

## Results

### BECLIN-1 loss leads to mislocalization of OCCLUDIN that mirrors E-CADHERIN mislocalization in intestinal organoids

Our published studies showed that BECLIN-1 deletion in intestinal organoids leads to the mislocalization of E-CADHERIN at the AJ, which we hypothesized contributes to increased intestinal epithelial permeability in mice [11]. However, in addition to AJs, epithelial barrier integrity is maintained by the coordinated functions of several junctional complexes, including TJs and desmosomes [2]. Interestingly, a previous study demonstrated that while BECLIN-1 knockdown disrupted OCCLUDIN trafficking, it unexpectedly reduced TJ permeability in Caco-2 colon cancer cells grown in 2D cultures [13]. Given that our studies had suggested that BECLIN-1 loss compromises the epithelial barrier, we utilized our more physiologically relevant 3D small intestine-derived organoids to first investigate the role of BECLIN-1 in OCCLUDIN localization at TJs. These experiments used organoids at an earlier developmental stage (Day 5) compared to our previous work, allowing us to focus on events preceding overt BECLIN-1 deletion-induced organoid death. For clarity, references to the apical plasma membrane refer to the apicolateral junction, while the lateral membrane extends downward, stopping just before the basolateral junction (see Supplemental Figure S1 for details).

**Figure 1.**
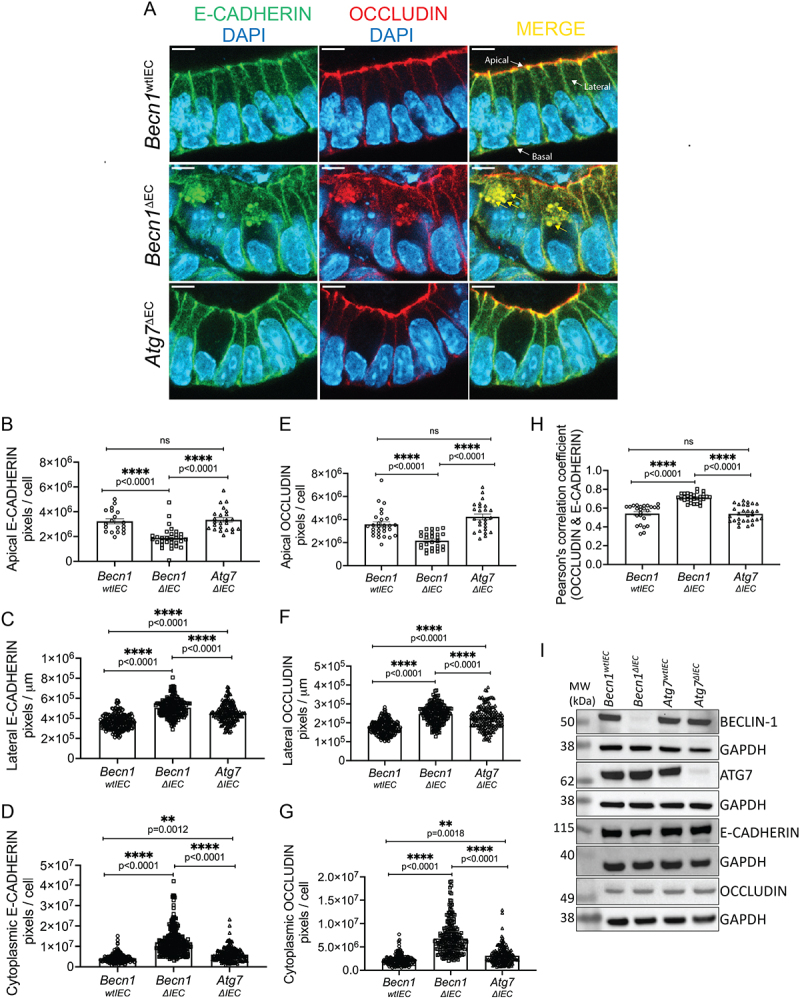
Mislocalization of E-CADHERIN and OCCLUDIN following BECLIN-1 loss in intestinal organoids.

As we showed previously [11], BECLIN-1 deletion led to a significant reduction in apicolateral E-CADHERIN localization in organoids derived from intestinal epithelial-specific BECLIN-1-deficient (*Becn1^∆IEC^*) mice compared to organoids derived from their wild-type littermates (*Becn1^wtIEC^*) (Figure 1A,B). This was accompanied by increased E-CADHERIN localization along the lateral membrane (Figure 1A,C) and enhanced cytoplasmic accumulation of E-CADHERIN (Figure 1A,D). OCCLUDIN, which is typically localized to the apicolateral region above E-CADHERIN [13], displayed a similar mislocalization pattern in *Becn1^∆IEC^* organoids. This included a significant reduction of apicolateral OCCLUDIN (Figure 1A,E), with increased lateral (Figure 1A,F) and cytoplasmic accumulation (Figure 1A,G), mirroring the changes seen with E-CADHERIN.

(A) Representative fields from whole-mount immunofluorescent staining of Day 5 post-4-HT-treated *Becn1*^wtIEC^, *Becn1*^ΔIEC^, and *Atg7^ΔIEC^* intestinal organoids. The absence of BECLIN-1, but not ATG7, leads to the mislocalization of OCCLUDIN, mirroring that of E-CADHERIN, as detected by immunofluorescence. The mislocalized junctional proteins appear as aberrantly enlarged cytoplasmic puncta, which co-localize with one another. Yellow arrows indicate representative enlarged cytoplasmic puncta. Labeled white arrows provide orientation of key membranes for all subsequent figures. Quantification of immunofluorescence staining shows (B) BECLIN-1 loss results in a significant reduction in apical membrane localization of E-CADHERIN with concurrent accumulation in (C) lateral membranes and (D) cytoplasm. These changes are mirrored in the (E) apical, (F) lateral, and (G) cytoplasmic localization of OCCLUDIN. (H) The loss of BECLIN-1, but not ATG7, also resulted in increased co-localization between E-CADHERIN and OCCLUDIN as measured using Pearson’s co-localization coefficient. (I) There was no change in total E-CADHERIN and OCCLUDIN protein levels between *Becn1*^ΔIEC^, Atg7^ΔIEC^, and their wild-type counterparts, as determined by Western blotting. GAPDH (glyceraldehyde-3-phosphate dehydrogenase) was used as a loading control for the blot directly above each GAPDH panel. Molecular weight (MW) markers indicate the relative size of the detected protein. Data are representative of at least *n = 3* biological replicates. Graphs show the mean ± S.E.M. Statistical significance was determined by ordinary one-way ANOVA for all comparisons. Scale bar = 10 µm.

Interestingly, deletion of ATG7 in intestinal organoids also caused mislocalization of both E-CADHERIN and OCCLUDIN, though these changes were less pronounced than those observed in *Becn1^∆IEC^* organoids (Figure 1A−G). In *Atg7^∆IEC^* organoids, the lateral and cytoplasmic accumulations of both proteins increased (Figure 1A,C,D,F,G), but apicolateral localization remained largely intact (Figure 1A,B,E), indicating that the significantly impaired apicolateral localization of these junctional proteins is unique to BECLIN-1 deletion.

Co-localization analysis further revealed a significantly higher overlap of E-CADHERIN and OCCLUDIN in *Becn1^∆IEC^* organoids (i.e. higher Pearson’s correlation coefficient) compared to both *Becn1^wtIEC^* and *Atg7^∆IEC^* organoids, suggesting abnormal accumulation of these proteins, most likely within defective RAB5A^+ve^ (Ras-related protein Rab-5a) early endosomes, as we previously reported [11] (Figure 1A,H). Importantly, no significant differences in the total cellular levels of E-CADHERIN and OCCLUDIN were observed across the genotypes (Figure 1I), suggesting that the observed mislocalization reflects trafficking defects rather than changes in expression levels.

In conclusion, the loss of BECLIN-1-mediated endocytic trafficking disrupts not only E-CADHERIN localization but also leads to similar mislocalization of OCCLUDIN. While ATG7 deletion also causes some mislocalization, it is less severe compared to BECLIN-1 loss, particularly with respect to apicolateral localization. These findings provide further insights into our previous observations where increased intestinal permeability was seen in *Becn1^wtIEC^* but not *Atg7^∆IEC^* mice [11].

### Dynamic assessment of endocytic trafficking in BECLIN-1- and ATG7-deleted mouse embryonic fibroblasts

Static imaging of RAB markers provides insights into endosomal distribution, but real-time analysis is crucial for understanding the dynamic nature of endocytic trafficking. To monitor cargo movement, live uptake assays were conducted using the pH-sensitive dye (pHrodo) conjugated to Dextran (bulk fluid-phase endocytosis) or Transferrin (receptor-mediated endocytosis), as well as AlexaFluor^TM^ 594-conjugated wheat germ agglutinin (WGA) (clathrin-dependent and independent pathways). For these experiments, SV40 large T antigen-transformed mouse embryonic fibroblasts (MEFs), derived from BECLIN-1 and ATG7 knockout mouse embryos, were utilized due to their suitability for live-cell imaging and their well-characterized membrane trafficking pathways [31]. Tamoxifen metabolite, 4-hydroxytamoxifen (4-HT) treatment resulted in successful deletion of BECLIN-1 (*Becn1^−/−^*) and ATG7 (*Atg7^−/−^*) at day 5 post-treatment (Supplemental Figure S2A). Similar to that observed in mouse IECs [11], *Becn1^−/−^* and *Atg7^−/−^* MEFs demonstrated defective basal autophagy flux, characterized by a pronounced accumulation of total SQSTM1/P62 (sequestosome 1) and MAP1LC3B (microtubule-associated protein 1 light chain 3 beta)-I and -II levels or a reduced MAP1LC3B-II:MAP1LC3B-I ratio compared to *Becn1^+/+^* and *Atg7^+/+^* controls (Supplemental Figure S2B).

Real-time imaging of *Becn1^−/−^* MEFs showed there was a significant reduction in Dextran- and WGA-positive puncta within the cytoplasm compared to wild-type controls and *Atg7^−/−^* MEFs (Supplemental Figure S2C−F). Instead, cargo strikingly accumulated near the plasma membrane following internalization (Supplemental Figure S2C), indicating a block in the early trafficking stages. Similarly, Transferrin uptake and trafficking *via* a more tightly regulated clathrin-dependent receptor-mediated endocytic process were also defective compared to wildtype (*Becn1^+/+^* and *Atg7^+/+^*) and *Atg7^−/−^* MEFs, with cargo stalling near the plasma membrane and failing to progress into endosomes (Supplemental Figure S2E,G).

These results suggest that BECLIN-1, unlike ATG7, is critical for the proper progression of cargo through the early endocytic stages. The disruption across different pathways (i.e. fluid-phase, receptor-mediated, and clathrin-independent) indicates a potential common point of failure at the level of the RAB5A^+ve^ early endosome, consistent with the effect of BECLIN-1 deletion on this compartment in our recent studies [11].

### BECLIN-1 deletion impairs cargo trafficking for lysosomal degradation

Given BECLIN-1ʹs critical role in early endosomal trafficking, distinct from ATG7, we next examined its influence on lysosomal function to elucidate further the broader implications of its deletion in cargo degradation processes. Lysosomal dysfunction could provide a mechanistic link between the observed mislocalization of junctional proteins and defective degradation pathways, contributing to the cellular stress seen in BECLIN-1-deficient organoids [11].

Here, we used LysoTracker Red^TM^ DND-99 (subsequently referred to as LysoTracker Red) to label acidic organelles (such as lysosomes) and pHrodo-Dextran, which fluoresces more intensely in acidic environments. Fluorescently tagged Dextrans have been observed to be trafficked to the lysosomes from as early as 780 s (13 min) following incubation and accumulate in the lysosomes without degradation for at least 24–72 h, enabling tracking of cargo to the lysosomes [32].

**Figure 2.**
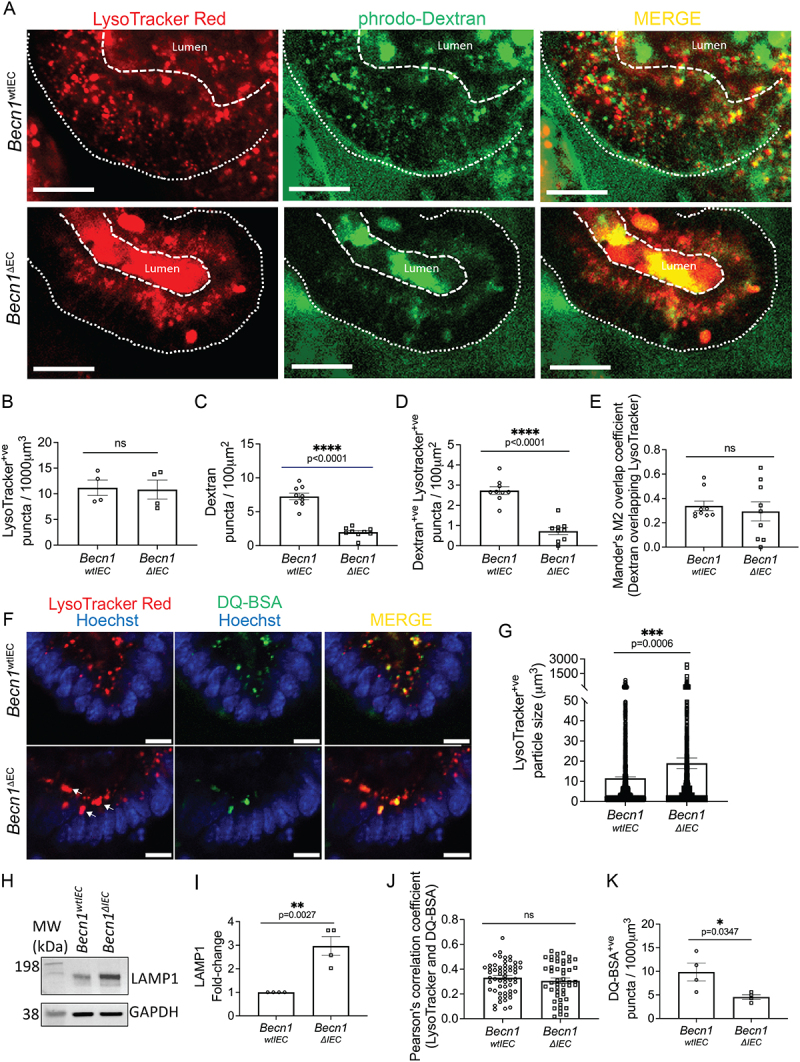
BECLIN-1 loss impairs cargo trafficking to the lysosomes.

At day 5 post-gene deletion, lysosome numbers measured by LysoTracker Red puncta were unchanged across *Becn1^wtIEC^* and *Becn1^∆IEC^* organoids (Figure 2A,B), however *Becn1^∆IEC^* organoids displayed reduced pHrodo-Dextran puncta (Figure 2C) and fewer pHrodo-Dextran^+ve^/LysoTracker Red^+ve^ puncta (Figure 2D), indicating impaired cargo trafficking to the lysosomes. However, the proportion of pHrodo-Dextran puncta overlapping LysoTracker Red (as measured by Manders’ M2 coefficient) was unchanged (Figure 2E), suggesting that cargo-lysosome fusion is still functional.

(A) Representative image of live intestinal organoids showing uptake of LysoTracker Red (red, marking lysosomes) and pHrodo-Dextran (green, a marker of fluid-phase endocytosis), 3 h post-incubation. Quantification of these data revealed (B) no significant change in lysosome numbers, and (C) a reduction in Dextran puncta following BECLIN-1 loss. (D) Additionally, there was a significant reduction in Dextran^+ve^LysoTracker Red^+ve^ puncta in *Becn1^∆IEC^* organoids, (E) but no change in their co-localization, as quantified using Mander’s overlap coefficient. (F) Representative fields from live imaging of intestinal organoids at Day 5 post-4-HT treatment using Lysotracker Red (red, marking lysosomes) and DQ-BSA (green, a marker of cargo degradation). Arrows indicate examples of enlarged lysosomes. (G) Quantification of >1900 puncta revealed an increase in Lysotracker Red puncta size. (H) Similarly, increased LAMP1 levels in *Becn1^∆IEC^* organoids were detected *via* Western blotting. GAPDH was used as a loading control. A representative example is shown. Molecular weight (MW) markers indicate the relative size of the detected proteins. (I) The fold-change in LAMP1 levels following BECLIN-1 deletion, as determined by Western blot analysis from n = 4 biological replicates. (J) Co-localization analysis between LysoTracker Red and DQ-BSA, as measured by Pearson’s correlation coefficient, revealed no significant changes between *Becn1^wtIEC^* and *Becn1^∆IEC^* organoids though (K) there were reduced DQ-BSA puncta following BECLIN-1 loss. Graphs show the mean ± S.E.M. Significance was determined by Student’s (unpaired) t-test. Scale bar = 10 µm.

Additionally, *Becn1^∆IEC^* organoids exhibited significantly enlarged lysosomes (i.e. LysoTracker Red^+ve^ puncta) and a 3-fold increased (*p* = 0.0027) level of the lysosomal membrane protein, LAMP1 (lysosomal-associated membrane protein 1) (Figure 2A,F−I). To further assess lysosomal function, organoids were treated with Dequenched Green Bovine Serum Albumin (DQ^TM^ Green BSA, referred to as DQ-BSA), a fluorescent cargo that emits fluorescence upon degradation in the lysosomes. DQ-BSA fluorescence remained detectable in enlarged lysosomes in *Becn1^∆IEC^* organoids (Figure 2F), with no significant change in the co-localization between LysoTracker Red and DQ-BSA (Figure 2J), indicating that lysosomes retain the ability to degrade cargo. However, *Becn1^∆IEC^* organoids showed a significant reduction in DQ-BSA puncta compared to *Becn1^wtIEC^* organoids (Figure 2K), suggesting an overall decrease in the amount of cargo being degraded.

Together, these findings indicate that although cargo reaching lysosomes can still undergo degradation, defective trafficking limits the overall efficiency of lysosomal activity in *Becn1^∆IEC^* organoids.

### Disruption of ACTIN cytoskeleton integrity in intestinal organoids upon BECLIN-1, but not ATG7, deletion

Having established that BECLIN-1, but not ATG7, loss disrupts endocytic trafficking and the correct localization of junctional proteins such as E-CADHERIN and OCCLUDIN, we next investigated the F-actin cytoskeleton. F-actin filaments are fundamental to maintaining intestinal epithelial structure and function, including junction assembly, stability, and endocytic trafficking [33]. The cortical actin network, particularly the apical F-actin belt, is directly associated with TJs and AJs, and the correct localization of TJs and AJs is important for the formation of the F-actin cytoskeleton. Additionally, F-actin filaments play a key role in the assembly and sorting of tubular carriers as well as endosome maturation and movement [33]. Given the interconnected roles of the F-actin cytoskeleton in junctional protein localization and trafficking, we hypothesized that BECLIN-1 loss might also affect F-actin organization, contributing to the observed disruption in junctional protein integrity and barrier function.

**Figure 3.**
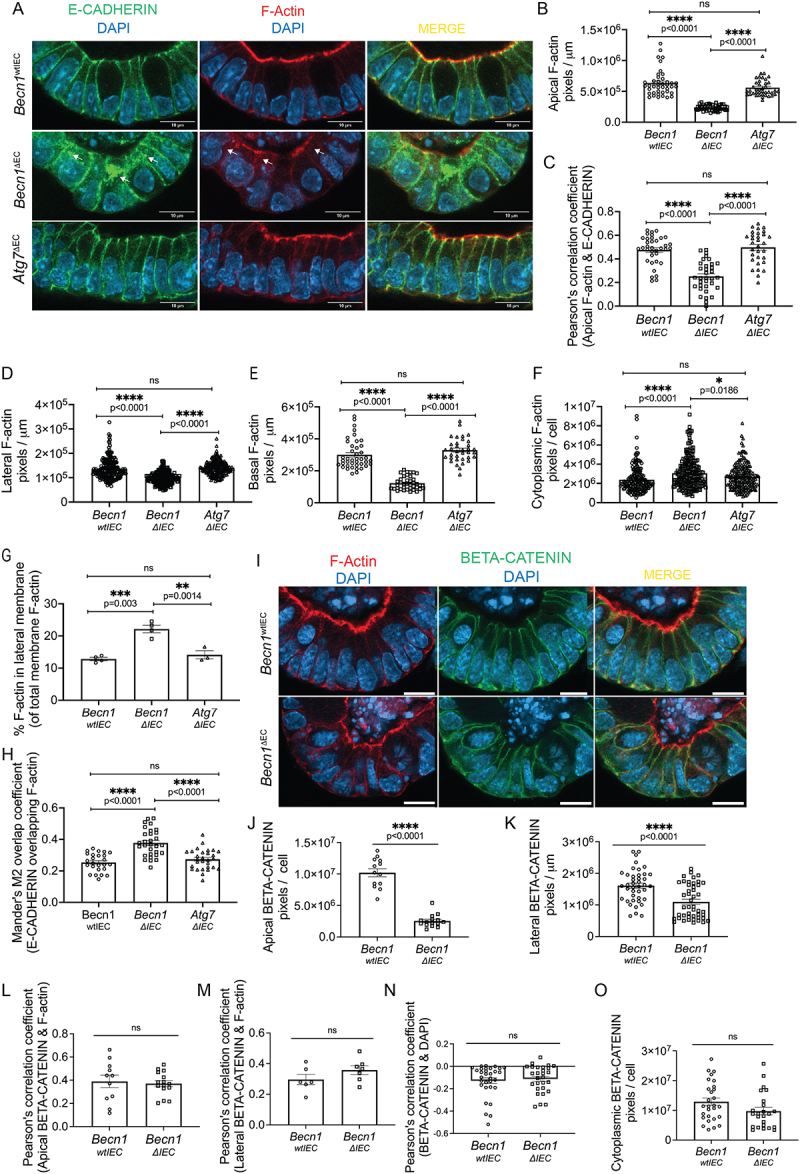
Disruption of the F-actin cytoskeleton following BECLIN-1 loss.

Whole-mount staining of intestinal organoids for F-actin and E-CADHERIN revealed a marked reduction in the apical F-actin belt in *Becn1^∆IEC^* organoids compared to *Becn1^wtIEC^* and *Atg7^∆IEC^* organoids (Figure 3A,B). This reduced F-actin belt was accompanied by decreased co-localization of F-actin with E-CADHERIN at apicolateral junctions, as evidenced by Pearson’s correlation coefficient (Figure 3A,C).

(A) Representative fields from whole-mount staining of intestinal organoids with E-CADHERIN and F-actin. Arrows indicate regions of mislocalized E-CADHERIN or F-actin. Quantification of these data revealed (B) significant depletion of apical F-actin staining and (C) reduced co-localization of E-CADHERIN and F-actin at the apicolateral junction, as measured by Pearson’s correlation coefficient, following BECLIN-1 loss. Additionally, (D) lateral and (E) basal F-actin expression in intestinal organoids was significantly decreased, while (F) cytoplasmic accumulation of F-actin was evident. (G) BECLIN-1 loss led to an increased ratio of lateral F-actin relative to total membrane-associated F-actin and (H) increased overlap of E-CADHERIN and F-actin at the lateral membrane and cytoplasm. Manders’ M2 coefficient was used to specifically quantify the proportion of E-CADHERIN (Channel B) overlapping F-actin (Channel A). (I) Representative fields from whole-mount immunostaining of F-actin and BETA-CATENIN also revealed a significant decrease in (J) apical and (K) lateral membrane staining of BETA-CATENIN following BECLIN-1 loss, with (L, M) no changes in the co-localization of F-actin and BETA-CATENIN in both regions. (N) There were also no significant changes in the co-localization of BETA-CATENIN with DAPI and (O) no increase in BETA-CATENIN cytoplasmic accumulation following BECLIN-1 loss. Data were representative of at least *n = 3* biological replicates. Graphs show the mean ± S.E.M. Statistical significance was determined by ordinary one-way ANOVA for B-H and Student’s unpaired t-test for J-O. Scale bar = 10 µm.

Further analysis revealed that *Becn1^∆IEC^* organoids also had a significant reduction in lateral and basal F-actin, associated with increased cytoplasmic accumulation of F-actin compared to controls (Figure 3D−F). However, there was a redistribution of F-actin toward the lateral membrane as indicated by the increased ratio of lateral membrane-associated F-actin relative to total membrane-associated F-actin (Figure 3G). This increased lateral and cytoplasmic F-actin overlapped (Figure 3H) with an increase in lateral and cytoplasmic E-CADHERIN in *Becn1^∆IEC^* organoids (Figure 1C,D), which was not observed in *Becn1^wtIEC^* and *Atg7^∆IEC^* organoids.

These findings demonstrate that BECLIN-1 deletion alters F-actin organization in intestinal organoids, which is critical for maintaining epithelial structure and integrity. The disruption of the F-actin cytoskeleton would not only exacerbate epithelial barrier dysfunction, but it would also contribute to impairment in endocytic trafficking, leading to the destabilization of cell junctions.

### Reduction of F-actin in *Becn1^∆IEC^* organoids is not attributed to defects in the BETA-CATENIN-F-actin interaction

The recruitment and organization of F-actin at junctional sites are partially regulated by BETA-CATENIN, a key component of the Wnt signaling pathway that interacts with E-CADHERIN [34]. As BETA-CATENIN mediates cytoskeletal remodeling and junction integrity by forming part of the multiprotein complex with CATENIN ALPHA 1 that links E-CADHERIN to F-actin, we investigated whether the reduction of F-actin in *Becn1^∆IEC^* organoids is associated with changes in junctional BETA-CATENIN. Additionally, BETA-CATENIN plays a dual role in Wnt signaling, translocating to the nucleus to regulate gene expression related to the cytoskeletal organization. Hence, we also examined whether BECLIN1 loss affected BETA-CATENIN localization or its nuclear translocation.

Whole-mount staining showed a significant reduction in apical and lateral BETA-CATENIN signals in *Becn1^∆IEC^* organoids compared to controls (Figure 3I−K). This reduction mirrored the decreased F-actin in these regions (Figure 3B,D). However, despite the decrease in BETA-CATENIN and F-actin levels, there was no significant difference in their co-localization between *Becn1^∆IEC^* and *Becn1^wtIEC^* organoids (Figure 3I,L,M). This suggests that BECLIN-1 loss affects F-actin levels and BETA-CATENIN distribution without altering their spatial association.

To investigate whether the reduction of BETA-CATENIN from the membrane was associated with increased nuclear translocation, we assessed BETA-CATENIN localization in the nucleus. No significant increase in nuclear BETA-CATENIN was observed between *Becn1^∆IEC^* and *Becn1^wtIEC^* organoids (Figure 3I,N). Additionally, there was no evidence of cytoplasmic accumulation of BETA-CATENIN, suggesting that normal degradation processes involved in BETA-CATENIN turnover of excess protein (i.e. BETA-CATENIN not bound to E-CADHERIN or WNT signaling) are not affected [35] (Figure 3I,O).

Combined, these data indicate that the disruption of the F-actin cytoskeleton is not due to impairments in BETA-CATENIN-mediated pathways, such as its role in linking E-CADHERIN to F-actin or in Wnt signaling. Instead, it suggests that BECLIN-1 influences F-actin organization through alternative mechanisms, highlighting its unique contribution to maintaining epithelial integrity.

## Discussion

The intestinal epithelium forms a critical barrier that regulates the selective passage of substances while maintaining tissue integrity, which is essential for proper physiological function and protection against pathogens [2]. This barrier is maintained by an intricate network of junctional complexes, including AJs and TJs, that ensure cell–cell adhesion and control paracellular permeability [36]. The AJ is also linked to the ACTIN cytoskeleton *via* complexes involving proteins such as CATENIN ALPHA-1 and BETA-CATENIN [33]. Importantly, epithelial tissues undergo constant remodeling to maintain their integrity in the face of dynamic environmental conditions and cellular stressors. This remodeling involves the coordinated trafficking of junctional proteins and the reorganization of the cytoskeleton, both of which are essential for preserving tissue architecture and barrier function. In this current report, we have significantly expanded on our previous studies to provide a more comprehensive understanding of how BECLIN-1 loss impacts these interconnected processes associated with maintaining epithelial integrity (see Supplemental Figure S1 for a schematic integrating all these new data).

BECLIN-1 is a key regulator of endocytic trafficking, primarily through its role in phosphatidylinositol 3-phosphate (PtdIns3P) production, which is essential for the maturation of endosomal compartments and the fusion of endosomes with lysosomes [37]. Loss of BECLIN-1 likely reduces PtdIns3P availability on endosomal membranes, leading to altered membrane dynamics and the accumulation of dysfunctional vesicular compartments [11]. In addition to E-CADHERIN [11], we now show that BECLIN-1 loss leads to significant mislocalization of OCCLUDIN, resulting in their accumulation in the cytoplasm rather than their proper localization at cell junctions. This disruption is likely to reflect a failure in the endocytic trafficking machinery, where internalized proteins are no longer efficiently routed through early endosomes for recycling or degradation [38]. We previously identified that BECLIN-1-deficient cells exhibit defective RAB5A^+ve^ and early endosome maturation, impairing E-CADHERIN trafficking. Here, live-cell imaging studies in MEFs further revealed that BECLIN-1 loss results in cargoes like Dextran and Transferrin accumulating near the plasma membrane and failing to progress through the endosomal pathway, implying a defect downstream of the internalization process. Additionally, some studies have demonstrated that lysosomal activity linked to autophagy contributes to cargo degradation *via* RAB7^+ve^ vesicles [30,39]. Notably, we previously showed that the RAB7^+ve^ vesicles were smaller in size following ATG7 loss [11], which would not only disrupt RAB7-mediated cargo degradation pathways but also impair autophagy-associated lysosomal degradation. Disruption in E-CADHERIN and OCCLUDIN clearance would potentially explain why ATG7-deficient organoids also showed mislocalization of E-CADHERIN and OCCLUDIN.

Interestingly, a recent report showed that BECLIN-1 knockdown results in OCCLUDIN levels increasing on membranes in colorectal cancer cells (and decreasing when autophagy is induced using Tat-Beclin1 peptide) [13]. While this finding is at odds with our results, it is an inherently different experimental system employing 2D adherent cell cultures that preclude investigation of the natural crypt-villus architecture. Indeed, we do see the accumulation of OCCLUDIN on lateral membranes, but its correct location at the apicolateral junction is impaired, which cannot be as readily discerned in the 2D cell cultures used in that study. Moreover, the cells are of colonic origin and cancerous, hence epithelial remodeling and turnover may be quite different from those in normal IECs. As such, these factors complicate direct comparison with our system, which focused on normal (i.e. non-cancerous) small-intestine derived cells.

The connection between BECLIN-1 loss and E-CADHERIN mislocalization is further complicated when the process of E-CADHERIN turnover is considered. This normally involves its degradation in lysosomes following delivery by RAB7^+ve^ endosomes [40]. We previously noted that the number of RAB7^+ve^ endosomes is increased following BECLIN-1 loss [11], potentially as a compensatory mechanism in response to the increased levels of mislocalized E-CADHERIN. Furthermore, RAB7 overexpression has been shown to promote the formation of enlarged endolysosomes, which was observed in *Becn1^∆IEC^* organoids (Figure 2(A,F,G)), further disrupting normal trafficking and degradation processes [41]. Notably, in this current study, we demonstrated that cargo delivery to the lysosomes was impaired, which would further compound the apparent accumulation of E-CADHERIN in the cytoplasm.

The impact of E-CADHERIN mislocalization is of particular importance due to its downstream consequences with potential impact on the cytoskeleton. It is known that E-CADHERIN localization at the apicolateral membrane involves multiple pathways, including sorting and recycling *via* RAB5A^+ve^ and RAB11^+ve^ endosomes, respectively, and/or direct transport from the Golgi, which also involves a RAB11-dependent mechanism [38]. As BECLIN-1 loss leads to widespread alterations in the distribution of endosomal compartments, including RAB5A^+ve^ and RAB11^+ve^ endosomes, this likely accounts for the decreased apicolateral distribution of E-CADHERIN. Importantly, the process of “cadherin flow” can compensate for the reduction of E-CADHERIN at AJs by re-distributing E-CADHERIN from the lateral membrane toward the apical junction in an F-actin-dependent manner, thereby helping to re-establish cell–cell contacts [38,42]. The increased lateral localization of E-CADHERIN following BECLIN-1 loss suggests the activation of an alternate recycling pathway, potentially *via* RAB4-mediated rapid recycling, which bypasses the RAB11^+ve^ endosomal recycling pathway [38,43]. Cadherin flow also relies on the association of E-CADHERIN with F-actin *via* CATENIN ALPHA-1 and BETA-CATENIN, which was preserved following BECLIN-1 loss [42]. However, despite a compensatory increase in lateral F-actin, which suggests that cadherin flow may be enacted, this was insufficient to fully restore E-CADHERIN levels at the AJs.

The contractile tension generated at the cell junction, critical for transmitting mechanical forces between cells and preserving tissue integrity, relies on the interaction between F-actin and E-CADHERIN *via* the catenin complex composed of both CATENIN ALPHA-1 and BETA-CATENIN. Combined, these proteins contribute to the formation of the apical F-actin belt [33,36]. Of relevance to our model system, disruption of this F-actin belt has been associated with epithelial damage in models of colitis and in IBD patients [44–46]. Notably, this belt was reduced in the BECLIN-1 knockout organoids, which most probably contributes to the loss of GI tissue architecture both in the organoids and BECLIN-1-deficient mice. Mechanistically, this reduction of the F-actin belt is likely a consequence of E-CADHERIN reduction at the AJs, which recruits not only CATENIN ALPHA-1 and BETA-CATENIN for F-actin attachment but also proteins such as Rho effectors required for F-actin formation [47]. This is consistent with previous reports showing that BECLIN-1 interacts with the Rho GTPase, RAC1 (Rac family small GTPase 1), and BECLIN-1 loss leads to cytoskeletal defects in macrophages [48]. The reduction of E-CADHERIN at AJs likely also accounts for the reduced levels of BETA-CATENIN at the apical membranes.

Our data suggest that BECLIN-1 is pivotal in epithelial tissue remodeling, particularly in trafficking junctional proteins and reorganizing the cytoskeleton to ensure proper localization and degradation of key proteins like E-CADHERIN and OCCLUDIN (Supplemental Figure S1). Loss of BECLIN-1 disrupts epithelial remodeling, leading to impaired junctional integrity, increased permeability, and epithelial barrier dysfunction. These wide-ranging defects align with the gross and rapid disruption of the GI epithelial architecture observed in BECLIN-1-deficient mice. Hence, these findings highlight the importance of BECLIN-1 in epithelial homeostasis. While BECLIN-1 has not previously been implicated in diseases characterized by barrier defects, such as IBD, our findings raise the possibility of its involvement in such conditions, particularly those affecting the small bowel such as Crohn’s disease, given the severe enteritis observed following BECLIN-1 deletion. This highlights the need for further investigation into BECLIN-1ʹs role in epithelial homeostasis and disease.

## Methods

### Mice

Details of the *Becn1*^wtIEC^, Becn1*^∆IEC^*, *Atg7*^wtIEC^ and *Atg7*^∆IEC^ mice used for the generation of organoids and MEFs were described previously [11]. These were housed at the La Trobe Animal Research and Teaching Facility (LARTF, La Trobe University, VIC, Australia) under Specific Pathogen-Free conditions. All experiments performed were approved by the La Trobe University animal ethics committee (approvals AEC18024, AEC18036) in accordance with the *Australian code for the care and use of animals for scientific purposes*. We have complied with all relevant ethical regulations for animal use. In this study, organoids and MEFs derived from *Becn1^wtIEC^* mice were generally used throughout as wild-type controls as they are functionally equivalent to those from *Atg7^wtIEC^* [11].

### Intestinal organoid culture

Organoids were established from crypt-enriched fractions from the duodenum of untreated mice exactly as described previously [11]. Organoids were maintained at 37°C, 5% CO_2_, with media being replaced every 2–3 days. Passaging was performed every 7–10 days by mechanically dissociating organoids and re-seeding them into fresh Cultrex at a 1:3 split. To induce *Becn1* and *Atg7* deletion, organoids were seeded into media containing 200 nM 4-HT (Sigma-Aldrich, H7904) for 3 days and then maintained as per normal.

### Mouse embryonic fibroblast generation and culture

Mouse embryonic fibroblasts were generated from E13-E14.5 embryos derived from *Becn1*^wtIEC^, *Becn1*^∆IEC^, *Atg7*^wtIEC^, and *Atg7*^∆IEC^ mice and immortalized (at passage 2–4) with SV40 large T antigen, as described previously [49]. Cells were maintained in DME Kelso medium supplemented with 10% (v/v) fetal bovine serum, 250 mM L-asparagine, and 50 mM 2-mercaptoethanol. Deletion of *Becn1* and *Atg7* was achieved by culturing cells in the presence of 500 nM 4-HT for 3 days and then maintained as per normal. Protein deletion was confirmed by Western blot analysis.

### Western immunoblotting

Lysates of MEFs and organoids were prepared, electrophoresed, and transferred to membranes for Western blotting as described previously [11]. Following blocking in 5% (w/v) skim milk in PBS (2.7 mM KCl, 1.76  mM KH_2_PO_4_, 136.7 mM NaCl, 8.07 mM anhydrous Na_2_HPO_4_) for 1 h at room temperature (RT) with agitation, membranes were probed overnight at 4°C with the following antibodies at the following dilutions, prepared in 1% (w/v) skim milk in PBST (PBS + 0.05% (v/v) Tween-20): BECLIN-1, 1:500 (CST, 3495); ATG7, 1:500 (Sigma-Aldrich, A2856), SQSTM1/p62, 1:500 (CST, 5114); MAP1LC3B, 1:500 (Novus Biologicals, NB100-2220); E-CADHERIN, 1:600 (CST, 3195). LAMP1, 1:1000 (Abcam, ab24170); OCCLUDIN, 1:500 (Invitrogen, 33-1500); and GAPDH, 1:5000 (Invitrogen, MA5-15738). Membranes were washed in PBST and probed for 1 h at RT with the following antibodies, prepared in 1% (w/v) skim milk in PBST: Donkey anti-Rabbit IgG, 1:10,000 (GE Healthcare, NA943V), Goat anti-Mouse IgG, 1:10,000 (Sigma-Aldrich, A0168). Luminescent signals were visualized using the Western Lightning Plus-ECL (PerkinElmer) kit and the ChemiDoc Imaging System (Bio-Rad). Images were processed using the Image Lab (Bio-Rad).

### Intestinal organoid whole-mount immunofluorescence

The whole-mount intestinal organoid staining protocol is as described previously, which is an adaption of the methods from Dekkers, Alieva [50] with modifications to clearing and mounting steps. Briefly, organoids were removed from the matrix by incubation with ice-cold Gentle Cell Dissociation Reagent (STEMCELL Technologies,100-0485) with gentle rocking at 4°C for 60 min. Organoids were then fixed in 4% (w/v) paraformaldehyde solution (ProSciTech, C004) and blocked with Organoid Wash Buffer (DPBS (Gibco^TM^, 14190144) + 0.1% (w/v) TritonX-100 (Merck) + 0.2% (w/v) Bovine Serum Albumin (Merck, A3059)), followed by overnight incubation at 4°C with gentle rocking with the following primary antibodies at the indicated dilutions: E-CADHERIN, 1:400 (Invitrogen, 13-1900); OCCLUDIN, 1:100 (Invitrogen, 33-1500); F-actin, using 1X Phalloidin-iFluor 647 solution (Abcam, ab176759); BETA-CATENIN, 1:100 (Proteintech, 51067-2-AP). Following primary antibody incubation, organoids were washed extensively and incubated overnight at 4°C with gentle rocking using the following secondary antibodies: Goat anti-Rat IgG (H + L) cross-adsorbed secondary antibody, Alexa Fluor^TM^ 568 (1:400, Invitrogen, A- 11077); Goat anti-Mouse IgG (H + L) cross-adsorbed secondary antibody, Alexa Fluor^TM^ 488 (1:400, Invitrogen, A-11004); Goat anti-Rabbit IgG (H + L) Cross-Adsorbed Secondary Antibody, Alexa Fluor^TM^ 488 (1:400, Invitrogen, A-11008), and nuclear stain DAPI (1 μg/ml, Merck, D9542). Organoids were then subjected to another extensive washing step prior to sample clearing and mounting steps. Images were then acquired using a Zeiss LSM 980 with an Airyscan 2 confocal microscope. FastAiryscan 2 SR-4Y imaging mode was utilized, and parameters such as laser power and gain were kept consistent among samples. Images were taken using the 40x (water) objective with 1.7x Zoom and imaged once to avoid excessive photobleaching. z-stacks were acquired for each image at 5 µm intervals.

### Dynamic endocytic trafficking analysis

Mouse embryonic fibroblasts were seeded overnight in MEF culture media at 2000 cells/well in Nunc^TM^ Lab-Tek^TM^ II Chambered Coverglass slides (Thermo Scientific, 155409PK). To visualize the different trafficking pathways, the media was removed and the cells were washed with Live Cell Imaging Solution (LCIS) (Invitrogen, A59688DJ). The media was then replaced with LCIS containing either pHRodo-Dextran (for bulk fluid-phase endocytosis, 60 µg/ml, Invitrogen, P35368) or pHRodo-Transferrin (for receptor-mediated endocytosis, 60 µg/ml, Invitrogen, P35376) with WGA conjugated to AlexaFluor^TM^ 594 (for clathrin-dependent and independent pathways, 2 µg/ml, Invitrogen, W11262), along with Hoechst 33342 (2 µg/ml, ThermoFisher, 62249) to stain nucleic acids. After 30 min of incubation at 37°C, cells were imaged using a Zeiss LSM 980 confocal microscope under controlled conditions (37°C, 10% CO_2_), with z-stacks acquired at 2 µm intervals.

### Lysosome function studies

Intestinal organoids were cultured as described above. At day 5 post-4-HT treatment, organoids were carefully removed from Cultrex by performing a series of washes with ice-cold Advanced DMEM/F12 medium (Gibco, 12634010).

For DQ-BSA studies, organoids were then resuspended in a staining solution containing LCIS, LysoTracker Red^TM^ DND-99 (50 nM, Invitrogen^TM^, L7528), DQ^TM^ Green BSA (50 µg/ml Invitrogen^TM^, D12050), and Hoechst 33342 (5 µg/ml) and transferred to a Nunc^TM^ Lab-Tek^TM^ II Chambered Coverglass. The organoids were incubated in the staining solution for 1 h at 37°C in 5% CO_2._

For pHrodo-Dextran uptake studies, organoids were resuspended in a staining solution containing LCIS, LysoTracker Red^TM^ DND-99 (50 nM, Invitrogen^TM^, L7528), and pHRodo-Dextran (50 µg/ml, Invitrogen, P35368) in a Nunc^TM^ Lab-Tek^TM^ II Chambered Coverglass. The organoids were incubated in the staining solution for 3 h at 37°C in 5% CO_2_Following incubation, images were acquired using a Zeiss 980 Confocal Microscope at 40x magnification, with matched pinhole sizes for all lasers and z-stacks captured at 4 µm intervals.

### Quantitative image analysis

Fluorescent image analysis was performed using ImageJ software (Fiji). Spatial calibration was conducted by setting the appropriate scale for each image (Analyze > Set Scale). Quantification of apical, lateral, basal, and cytoplasmic fluorescence signals on whole-mount fluorescent images (Figures 1(A) and 3(A,I)) was conducted by manually drawing regions of interest (ROIs) around the relevant structures using the polygonal, freehand, and line selection tools and measured using the “Measure” (Analyze > Measure) function. A minimum of three ROIs (comprising >5 cells one after another) per stack, and at least three z-sections with clear apical-to-basal orientation, were analyzed per organoid.

For endocytic uptake (Supplemental Figure S2C,E), the z-stacks were compressed into a single layer using the Z-projection function (Image > Stacks > Z Project). ROIs were manually drawn around individual cells, with at least 10 cells analyzed per replicate. The fluorescence channel of interest was thresholded to generate a binary image (Image > Adjust > Threshold), followed by particle separation via watershed segmentation (Process > Binary > Watershed). Particles within the ROI were quantified using the Analyze Particle tool (Analyze>Analyze Particle).

For lysosome function analysis (Figure 2), fluorescent signals from each z-section were thresholded to isolate the structures of interest (i.e. LysoTracker Red DND-99^+ve^ puncta, pHrodo-Dextran, and DQ Green BSA^+ve^ puncta). Watershed segmentation (Process > Binary > Watershed) was performed next to separate puncta. For lysosome puncta number and size analysis, puncta were quantified using the 3D Object Counter tool (Analyze > 3D Objects Counter) to determine the number and the size of the puncta within the organoid z-stack. The number of puncta was normalized to the organoid volume. For Dextran puncta quantification, particle numbers were obtained using the Analyze Particle tool (Analyze>Analyze Particle), and the number of particles was normalized to the organoid area per z-slice.

Co-localization analysis was performed using the Just Another Co-localization Plugin (JaCoP; Bioimaging and Optics Platform, BIOP) with Otsu thresholding applied to define object boundaries. Co-localization analysis was performed using either Pearson’s correlation coefficient or Manders’ overlap coefficients (M1, M2), depending on the specific experimental objective. Pearson’s correlation was used to assess the overall spatial relationship between two markers when their distributions were expected to be coordinated but not necessarily overlapping, whereas Manders’ coefficients were applied when the aim was to quantify the fraction of one signal localized within another. Dextran^+ve^LysoTracker^+ve^ puncta quantification was obtained by measuring the number of overlapping puncta (Analyze>Analyze particle) following co-localization analysis.

### Statistics and reproducibility

Numerical source data for all graphs are provided in the “Methods” section and Figure legends. Statistical tests were performed using GraphPad Prism 8 Software (GraphPad, San Diego, CA) *via* Student’s unpaired t tests between two groups and one-way ANOVA (with Tukey post-hoc comparisons) for multiple comparisons. All data were obtained by performing at least n = 3 biological replicates with representative data shown and expressed as the mean ± standard error of the mean (S.E.M). P values < 0.05 were considered statistically significant. Significance levels were split further as follows: **P < 0.01, ***P < 0.001, ****P < 0.0001.

## Supplementary Material

Supplemental Material_Final_CLEAN.docx
