# Supplementary material for: BECLIN-1 is essential for the maintenance of gastrointestinal epithelial integrity by regulating endocytic trafficking, F-actin organization, and lysosomal function
Source: Autophagy Rep. 2025 Apr 3;4(1):2484494. doi: 10.1080/27694127.2025.2484494 (PMC11980461; doi:10.1080/27694127.2025.2484494)
Supplement: Supplemental Material_Final_CLEAN.docx [file KAUO_A_2484494_SM3366.docx]

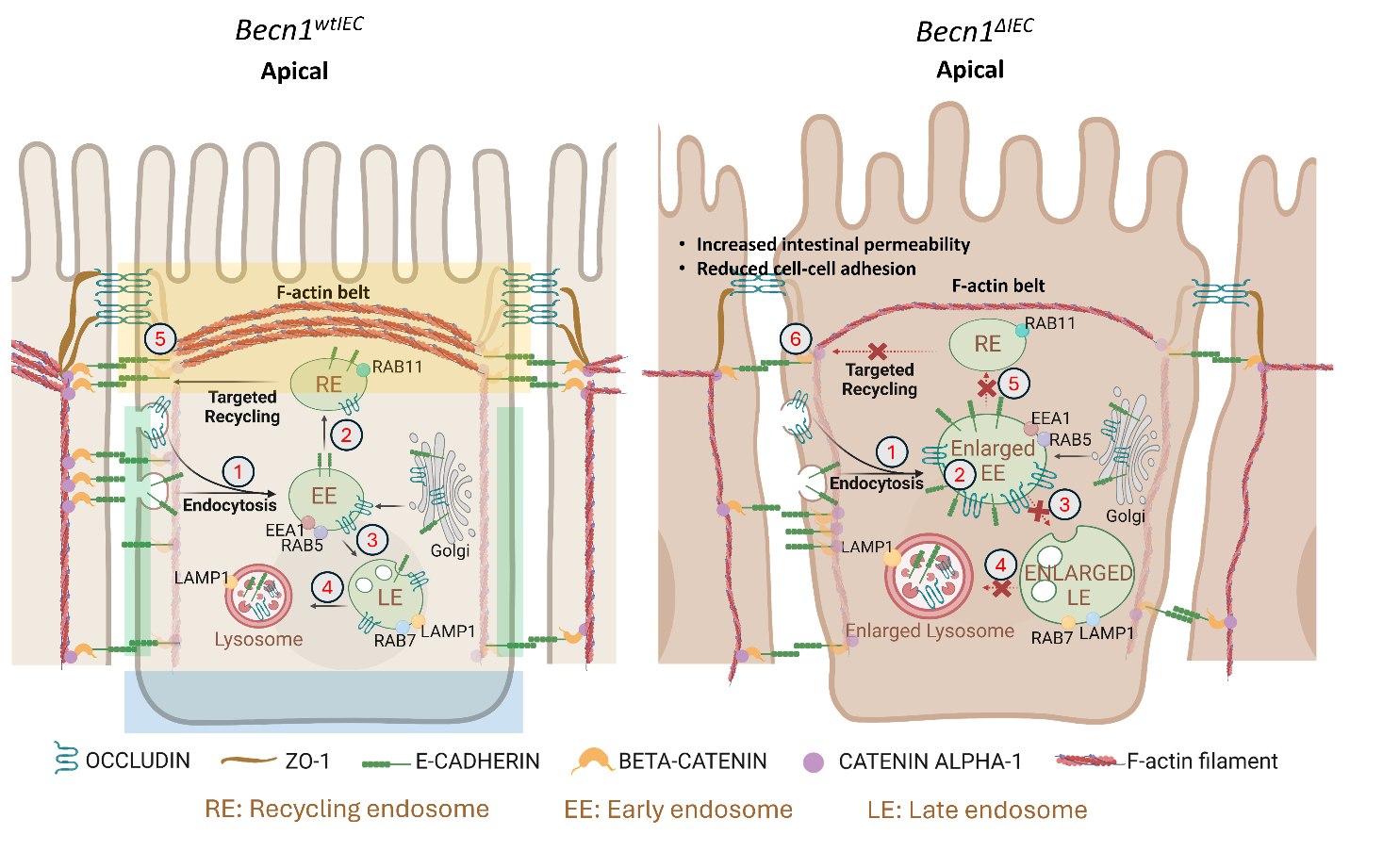


**Supplemental Figure S1.**

Schematic representing the BECLIN-1 loss impacts endocytosis, formation of cellular junctions and the cytoskeleton. **Left:** In healthy IECs undergoing epithelial remodelling, (1) membrane-bound E-CADHERIN and OCCLUDIN are internalised *via* endocytosis and transported to RAB5A^+ve^ early endosomes, where they undergo cargo sorting. The majority of E-CADHERIN and a portion of OCCLUDIN are (2) recycled back to the apicolateral junction through a RAB11-dependent targeted recycling pathway. (3) A large proportion of OCCLUDIN and some E-CADHERIN are sorted into RAB7^+ve^ late endosomes for (4) transport to the lysosomes, where they are degraded. (5) Once localized at the apicolateral junction, E-CADHERIN recruits F-actin polymerization machinery to form the cortical F-actin belt, generating the contractile tension essential for epithelial integrity. Orange, green and blue shaded boxes define the apical, lateral and basal domains referenced throughout the manuscript. **Right:** Upon BECLIN-1 loss, (1) internalized E-CADHERIN and OCCLUDIN are still transported to RAB5A^+ve^ early endosomes. However, due to defective endosomal maturation (RAB5A to RAB7 transition failure), (2) cargo accumulates in enlarged RAB5A^+ve^ early endosomes, (3) preventing further transport to RAB7^+ve^ late endosomes and (4) degradation in lysosomes. The late endosomes and lysosomes were also enlarged following BECLIN-1 loss. (5) Additionally, mislocalization of RAB11 in BECLIN-1-deficient IECs disrupts cargo sorting from RAB5A^+ve^ early endosomes to RAB11^+ve^ recycling endosomes, halting targeted recycling of cargoes to the apicolateral junction. (6) Reduced E-CADHERIN apicolateral localization impaired formation of the apical F-actin belt. Together, these trafficking defects lead to a significant reduction in E-CADHERIN and OCCLUDIN at the apicolateral junction, impairing F-actin polymerization and hindering the formation of the cortical F-actin belt, contributing to epithelial breakdown. Red dashed arrows with crosses denote defective or inefficient trafficking.


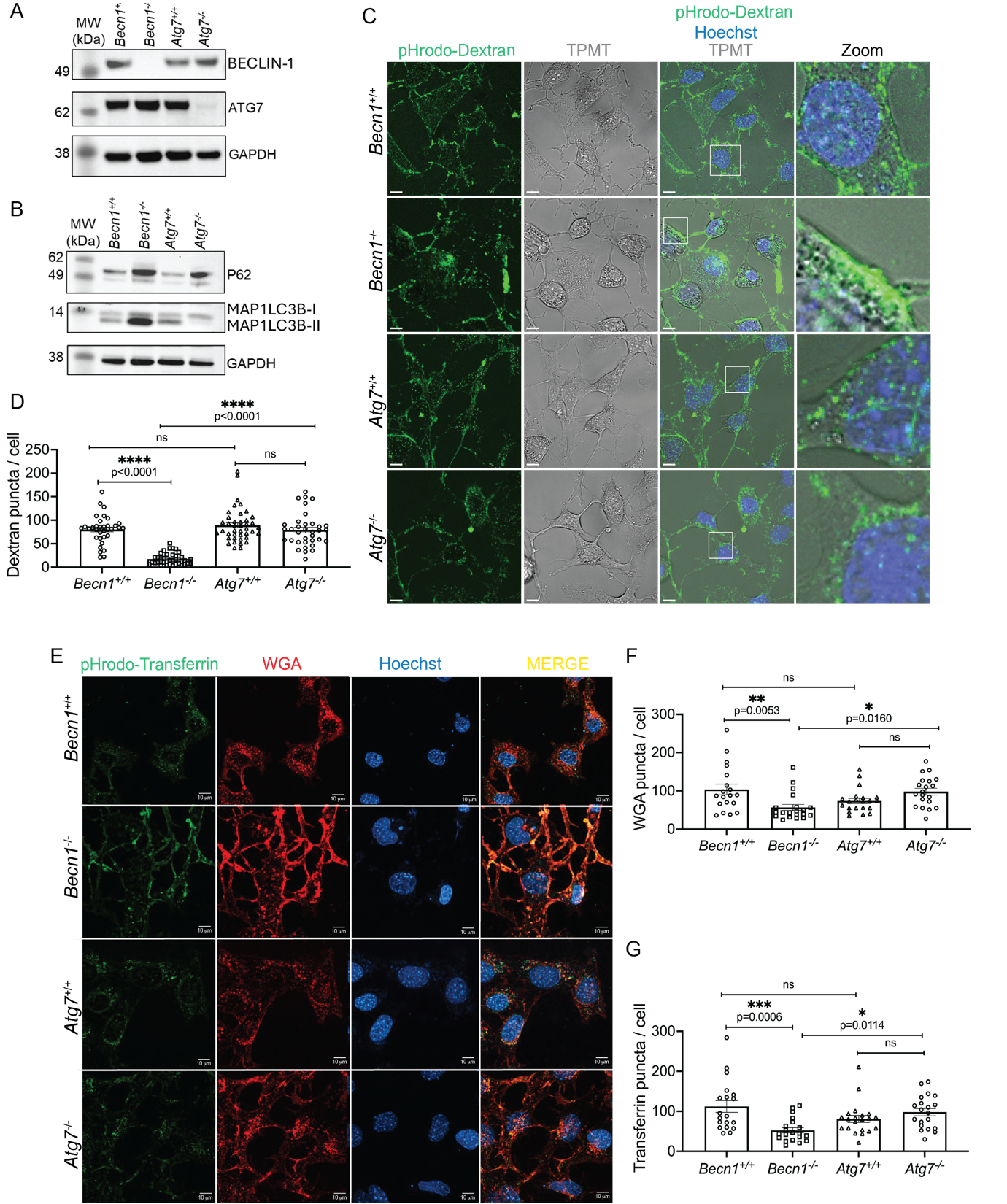


**Supplemental Figure S2. BECLIN-1 loss results in autophagy and endocytic trafficking defects in MEFs.**

**(A)** Deletion of BECLIN-1 and ATG7 at Day 5 post-4-HT in MEFs, determined by Western blotting. This led to (**B**) defective autophagy, indicated by increased levels of total P62 and MAP1LC3, or increased MAP1LC3-I relative to MAP1LC3-II. GAPDH was used as a loading control. Molecular weight (MW) markers indicate the relative size of the detected proteins. (**C**) Representative images of live pHrodo-Dextran uptake in MEFs, including two-photo microscopy transmission (TPMT) imaging, demonstrate the accumulation of mistraficked pHrodo-Dextran near the plasma membrane following BECLIN-1 loss. The white box is the region enlarged in the zoomed panels. **(D)** There were fewer pHrodo-Dextran puncta in the cytoplasm. This was also the case with **E, F)** wheat germ Agglutinin (WGA) and **G)** receptor-mediated endocytosis of Transferrin. Data were representative of at least *n =* 3 biological replicates. Graphs showed the mean ± S.E.M. Significance was determined by ordinary one-way ANOVA for all comparisons. Scale bar = 10 µm.
